# Effectiveness of icatibant for treatment of hereditary angioedema attacks is not affected by body weight: findings from the Icatibant Outcome Survey, a cohort observational study

**DOI:** 10.1186/s13601-018-0195-x

**Published:** 2018-03-23

**Authors:** Teresa Caballero, Andrea Zanichelli, Werner Aberer, Marcus Maurer, Hilary J. Longhurst, Laurence Bouillet, Irmgard Andresen, the IOS Study Group, W. Aberer, M. Wiednig, A. Grumach, A. Bygum, C. Blanchard Delaunay, I. Boccon-Gibod, L. Bouillet, B. Coppere, O. Fain, B. Goichot, A. Gompel, S. Guez, P. Y. Jeandel, G. Kanny, D. Launay, H. Maillard, L. Martin, A. Masseau, Y. Ollivier, A. Sobel, J. Arnolds, E. Aygören-Pürsün, M. Baş, M. Bauer, K. Bork, I. Martinez-Saguer, M. Maurer, E. Papadopoulou-Alataki, F. Psarros, Y. Graif, S. Kivity, A. Reshef, E. Toubi, F. Arcoleo, M. Bova, M. Cicardi, P. Manconi, G. Marone, V. Montinaro, A. Zanichelli, M. L. Baeza, T. Caballero, R. Cabañas, M. Guilarte, D. Hernandez, C. Hernando de Larramendi, R. Lleonart, T. Lobera, L. Marques, B. Saenz de San Pedro, J. Björkander, C. Bethune, T. Garcez Pereira, M. Helbert, H. J. Longhurst

**Affiliations:** 1 Allergy Department, Hospital La Paz Institute for Health Research (IdiPaz), Biomedical Research Network on Rare Diseases (CIBERER, U754), Madrid, Spain; 2 Department of Biomedical and Clinical Sciences Luigi Sacco, University of Milan, ASST Fatebenefratelli Sacco, Milan, Italy; 3 Department of Dermatology and Venerology, Medical University of Graz, Graz, Austria; 4 Department of Dermatology and Allergy, Charité – Universitätsmedizin Berlin, Berlin, Germany; 5 Department of Immunology, Barts Health NHS Trust, London, UK; 6 National Reference Centre for Angioedema, Internal Medicine, Grenoble University Hospital, Grenoble, France; 7 Shire, Zug, Switzerland; 8 Servicio de Alergia, Hospital Universitario La Paz, Paseo de la Castellana 261, 28046 Madrid, Spain; 9 Present Address: Addenbrooke’s Hospital, Cambridge University Hospitals NHS Foundation Trust, Cambridge, UK

**Keywords:** Hereditary angioedema, Icatibant, Bradykinin, Body mass index

## Abstract

**Background:**

Icatibant is a bradykinin B2-receptor antagonist used for the treatment of hereditary angioedema attacks resulting from C1-inhibitor deficiency. Treatment is not adjusted by body weight however the impact of body mass index (BMI) on the effectiveness of icatibant is not documented in the literature. We examined disease characteristics and icatibant treatment effectiveness in patients stratified by BMI in the Icatibant Outcome Survey, an ongoing, international, observational study monitoring the real-world safety and effectiveness of icatibant.

**Methods:**

Attack and treatment characteristics as well as outcomes following treatment with icatibant were compared among patients with underweight, normal, overweight, and obese BMI.

**Results:**

Data from 2697 icatibant-treated attacks in 342 patients (3.5, 44.7, 34.8, and 17.0% patients of underweight, normal, overweight, and obese BMI, respectively) were analyzed. There was no significant difference in the frequency and severity of attacks across BMI groups, although obese patients tended to have more attacks of high severity. There was no impact of BMI on the frequency of laryngeal attacks, but patients with normal BMI had fewer cutaneous attacks and more abdominal attacks. Most attacks (71.9–83.8%) were treated with a single icatibant injection without the need for rescue with plasma-derived C1-inhibitor (pdC1-INH), regardless of BMI. Patients with obese BMI used pdC1-INH as rescue treatment more often (P < 0.0001; P = 0.0232 excluding 2 outliers) and treated attacks earlier than patients with normal BMI (P = 0.007). Furthermore, time to resolution and duration of attack were shorter for patients with high BMI (P < 0.001 for overweight and P < 0.05 for obese versus normal).

**Conclusion:**

Overall, icatibant was comparatively effective in treating attacks in patients across all BMI groups.

*Trial registration* NCT01034969.

**Electronic supplementary material:**

The online version of this article (10.1186/s13601-018-0195-x) contains supplementary material, which is available to authorized users.

## Background

Hereditary angioedema due to C1-inhibitor (C1-INH) deficiency (C1-INH-HAE) is a genetic disease, affecting one in 50,000 [1] people, with symptoms such as localized cutaneous swelling, abdominal pain, and laryngeal edema [2]. C1-INH-HAE is caused by mutations in the *SERPING1* gene, leading to C1-INH deficiency and subsequently elevated levels of bradykinin, the mediator of increased vascular permeability during attacks [1, 3].

Icatibant (Firazyr^®^; Shire, Zug, Switzerland) is a subcutaneously administered bradykinin B2 receptor antagonist that has demonstrated efficacy and safety for the treatment of acute attacks of C1-INH-HAE [4, 5]. The approved dose of icatibant in patients ≥ 18 years of age is 30 mg, and is based on clinical studies using a dose of 0.4 mg/kg body weight. Clinical trials did not show an impact of body weight on the safety and efficacy of icatibant, and dosing is not adjusted by body weight. However, the effect of body weight on treatment outcomes has not been evaluated in the real-world setting. Body weight can have an impact on the pharmacokinetics and pharmacodynamics of a drug and, subsequently, its effectiveness. In addition, the relationship between body weight and the characteristics of C1-INH-HAE attacks is not known.

The Icatibant Outcome Survey (IOS; NCT01034969) is an ongoing international observational study that monitors the safety and effectiveness of treatment with icatibant. In this analysis, the relationship between body mass index (BMI), attack characteristics, and icatibant treatment outcomes in patients with C1-INH-HAE type I and type II receiving icatibant was investigated in the real-world setting.

## Methods

### Study design

Details on the design and conduct of IOS are described elsewhere [6]. Patients were enrolled from 51 sites in 11 countries: Brazil, Israel, and across Europe. In this analysis, data from icatibant-treated patients with C1-INH-HAE type I or II were obtained from patients who entered the study between July 2009 and February 2016. Patients were divided into groups according to their BMI at baseline (i.e., before enrollment): underweight (< 18.5 kg/m^2^), normal (18.5 to < 25.0 kg/m^2^), overweight (25.0 to < 30.0 kg/m^2^), or obese (≥ 30 kg/m^2^). Details regarding the characteristics of icatibant-treated C1-INH-HAE attacks and the use of any concomitant or rescue medications, including C1-INH, were collected via physician-completed electronic forms at routine visits (recommended every 6 months). Patients were educated by their HAE specialist to report attack severity based on the extent of interference with daily activities. Attack severity was classified as: very mild (very mild interference with daily activities); mild (mild interference with daily activities); moderate (moderate interference with daily activities and no other countermeasures required); severe (severe interference with daily activities and with or without other countermeasures); or very severe (very severe interference with daily activities and other countermeasures required).

### Statistical analysis

Due to the small number of patients and attacks in the underweight BMI group, statistical comparisons of BMI groups did not include the underweight category, and the results of the underweight BMI group are only summarized descriptively. Statistical testing was considered exploratory in this observational study and no adjustment for multiplicity was performed. There was no imputation of data from patients who discontinued from the study.

Attack rate and duration of untreated attacks were both compared using the Kruskal–Wallis test. Attack severity, attack site, and the use of plasma-derived C1-INH as a rescue medication in the normal, overweight, and obese BMI groups were compared using a generalized linear model of repeated measures (PROC GLIMMIX; SAS Institute Inc., Cary, NC, USA).

Treatment outcomes included time to treatment (time from attack onset to icatibant injection), time to resolution (time from icatibant injection to complete symptom resolution), and attack duration (time from attack onset to complete resolution of symptoms). A mixed-model analysis of repeated measures (PROC MIXED; SAS Institute Inc.) was used to compare mean time to treatment, time to resolution, and duration of attack data for patients in the BMI groups using base-10 log-transformed time data (h). The impact of BMI along with sex, age (i.e., factors that influence BMI), and other patient and attack characteristics on treatment outcomes were analyzed using a generalized linear model of repeated measures with PROC GENMOD (SAS Institute Inc.). A multivariate model was built using a backward selection process, which incorporated variables from the univariate model with P values < 0.20 and removed factors with the highest P values until only significant factors remained (P ≤ 0.05). Odds ratios (ORs) and corresponding 95% confidence intervals were estimated.

## Results

### Patient characteristics

The analysis included data from 2697 icatibant-treated attacks reported by 342 patients with C1-INH-HAE for whom baseline BMI data were available. Of the 342 patients, 12 (3.5%), 153 (44.7%), 119 (34.8%), and 58 (17.0%) had an underweight, normal, overweight, and obese BMI, respectively (Table 1). There was a comparable distribution of males and females among patients with overweight or obese BMI, but most patients with normal or underweight BMI were female. Almost half (n = 169; 49.4%) of the patients were using long-term prophylaxis. There was no difference among the normal, overweight, and obese BMI groups in the type of long-term prophylaxis therapy used.

**Table 1:** Patient demographics and number of icatibant-treated attacks

Characteristic	Underweight BMI	Normal BMI	Overweight BMI	Obese BMI
Patients, n (%)	12 (3.5)	153 (44.7)	119 (34.8)	58 (17.0)
BMI (kg/m^2^)^a^				
Mean ± SD	18.0 ± 0.5	22.2 ± 1.8	26.9 ± 1.3	34.5 ± 4.1
Median (range)	18.1 (16.7–18.4)	22.4 (18.7–25.0)	26.6 (25.0–29.8)	33.3 (30.0–46.7)
Sex, n (%)				
Female	11 (91.7)	105 (68.6)	60 (50.4)	33 (56.9)
Male	1 (8.3)	48 (31.4)	59 (49.6)	25 (43.1)
Age at enrollment (years), n (%)				
≥ 12 to < 18	1 (8.3)	2 (1.3)	1 (0.8)	0
≥ 18 to < 30	8 (66.7)	53 (34.6)	24 (20.2)	10 (17.2)
≥ 30 to < 50	1 (8.3)	66 (43.1)	52 (43.7)	27 (46.6)
≥ 50 to < 65	1 (8.3)	27 (17.6)	30 (25.2)	16 (27.6)
≥ 65	1 (8.3)	5 (3.3)	12 (10.1)	5 (8.6)
Country, n (%)				
Austria	0	6 (3.9)	3 (2.5)	0
Brazil	0	6 (3.9)	7 (5.9)	3 (5.2)
Denmark	0	0	1 (0.8)	2 (3.4)
France	2 (16.7)	45 (29.4)	21 (17.6)	8 (13.8)
Germany	2 (16.7)	16 (10.5)	16 (13.4)	12 (20.7)
Greece	0	3 (2.0)	3 (2.5)	1 (1.7)
Israel	2 (16.7)	20 (13.1)	15 (12.6)	6 (10.3)
Italy	1 (8.3)	18 (11.8)	14 (11.8)	4 (6.9)
Spain	4 (33.3)	20 (13.1)	22 (18.5)	8 (13.8)
Sweden	0	0	1 (0.8)	0
United Kingdom	1 (8.3)	19 (12.4)	16 (13.4)	14 (24.1)
Ongoing long-term prophylaxis, n (%)				
n	3	75	58	33
C1-INH^b^	0	11 (14.7)	11 (19.0)	4 (12.1)
Attenuated androgens^b^	0	47 (62.7)	44 (75.9)	24 (72.7)
Tranexamic acid^b^	2 (66.7)	24 (32.0)	9 (15.5)	11 (33.3)
Other^b^	1 (33.3)	8 (10.7)	4 (6.9)	3 (9.1)
No. of icatibant-treated attacks during enrollment	104	1314	829	450
No. of icatibant-treated attacks per patient^c^				
Mean ± SD	8.7 ± 13.5	8.6 ± 14.8	7.0 ± 11.3	7.8 ± 10.8
Median (range)	4.0 (1–47)	4.0 (1–101)	3.0 (1–83)	3.5 (1–57)

*BMI* body mass index; *C1*-*INH* C1-inhibitor; *SD* standard deviation

^a^At study entry

^b^Percentage calculated from number of patients using long-term prophylaxis at study entry and/or during enrollment

^c^Attack rate during enrollment. P = 0.469 comparing the normal, overweight, and obese categories. The underweight category was excluded from the comparison due to small sample size. Two patients (one normal BMI, one obese BMI) were found to be outliers because of an abnormally high rate of reinjections and rescue medication use. When their data are excluded, mean ± SD for normal = 8.4 ± 14.6 attacks/patient and for obese = 6.9 ± 8.6 attacks/patient (Additional file 1: Table S1)

### Attack characteristics

**Fig. 1:**
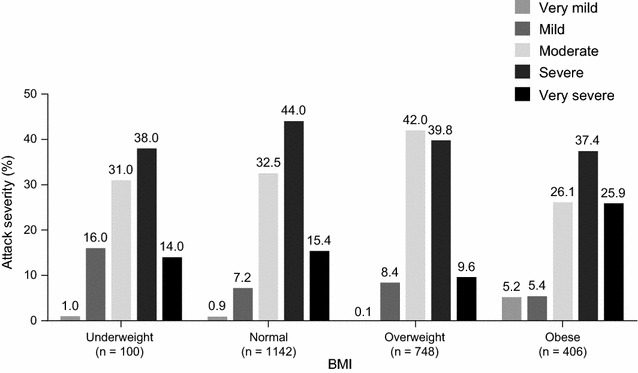
Severity of icatibant-treated attacks by body mass index (BMI). P values comparing severity of attacks (very mild/mild/moderate versus severe/very severe): P = 0.136 for normal versus overweight; P = 0.627 for normal versus obese; P = 0.109 for overweight versus obese. Results excluding data from the two reinjection outliers are presented in Additional file 1: Figure S1. n = number of attacks. Very mild = very mild interference with daily activities; mild = mild interference with daily activities; moderate = moderate interference with daily activities and no other countermeasures required; severe = severe interference with daily activities and with or without other countermeasures; very severe = very severe interference with daily activities and other countermeasures required

Among the normal, overweight, and obese BMI groups, there was no difference in the mean attack frequency per patient during enrollment (P = 0.469). There were more attacks classified as very severe in patients with obese BMI (25.9%) than in patients with normal (15.4%) or overweight (9.6%) BMI (Fig. 1); however, there were no statistically significant differences in attack severity within any of the groups (P > 0.1 comparing very mild/mild/moderate *versus* severe/very severe attacks).

**Fig. 2:**
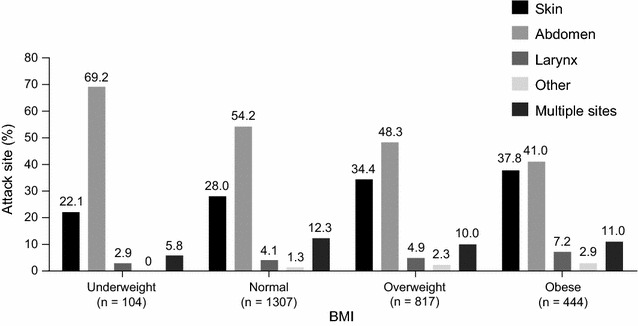
Site of icatibant-treated attacks by body mass index (BMI). P values comparing frequency of attacks between patients with normal/overweight/obese BMI: P = 0.020 for skin attacks; P = 0.003 for abdominal attacks; P = 0.282 for laryngeal attacks; P = 0.108 for attacks affecting other organs. Results excluding data from the two reinjection outliers are presented in Additional file 1: Figure S2. n = number of attacks

There were some significant differences in attack site frequency among the BMI groups: patients with normal BMI had fewer attacks affecting the skin (P = 0.020) and more attacks affecting the abdomen (P = 0.003; Fig. 2). There was no impact of BMI on the frequency of attacks affecting the larynx (P = 0.282).

The IOS database also captured some information on attacks that were untreated. A total of 309 patients reported the occurrence of untreated attacks at baseline, and 182 patients reported untreated attacks at follow-up (Table 2). When we compared the duration of untreated attacks across BMI groups, no statistical difference in the mean duration of attack among the three BMI groups was found (P = 0.408 at baseline, P = 0.530 at follow-up), although mean attack duration at follow-up tended to be longer in the obese BMI group.

**Table 2:** Average duration of untreated attacks

	Underweight BMI	Normal BMI	Overweight BMI	Obese BMI
*Baseline*				
Average duration of attack (h)				
n	12	150	106	41
Mean ± SD^a^	50.7 ± 22.9	40.5 ± 32.4	45.9 ± 31.5	44.4 ± 35.5
Median (range)	48.0 (6–92)	41.0 (0–140)	48.0 (0–156)	48.0 (0–120)
*Follow-up*				
Average duration of attack (h)				
n	11	76	70	25
Mean ± SD^a^	34.6 ± 21.6	39.7 ± 31.1	37.6 ± 30.6	44.2 ± 29.9
Median (range)	36 (0–72)	37.5 (0–144)	28.7 (0–120)	48.0 (0.3–120)

Average duration of untreated attacks corresponds to mean of average durations of untreated attacks at the skin, abdomen, larynx, and other sites

*BMI* body mass index; *SD* standard deviation; n = the number of patients

^a^P values comparing average duration of attack at baseline between patients with normal/overweight/obese BMI: P = 0.408 at baseline; P = 0.530 at follow-up. Results excluding data from the two reinjection outliers are presented in Additional file 1: Table S2

### Treatment characteristics

Icatibant use was comparable among the BMI groups (Table 3). More than 70% of icatibant injections among all BMI groups were self-administered. Overall, 88.3, 83.8, 83.2, and 71.9% of attacks in patients with underweight, normal, overweight, and obese BMI, respectively, were treated with a single icatibant injection and without plasma-derived C1-INH rescue medication. Two patients (one with normal BMI, one with obese BMI) were previously identified as outliers because of an abnormally high rate of reinjections and rescue medication use [7]. Patient characteristics excluding the outliers are presented in Additional file 1: Table S1. When data from the two outliers were excluded, 88.3, 84.6, 83.2, and 82.0% of attacks in patients with underweight, normal, overweight, and obese BMI, respectively, were treated with a single icatibant injection (Additional file 1: Table S3). A single dose of plasma-derived C1-INH (pdC1-INH) was administered as rescue medication in 8.7, 7.7, 10.9, and 21.3% of attacks for patients with underweight, normal, overweight, and obese BMI, respectively (*P* < 0.0001). In most attacks that were treated with pdC1-INH rescue, pdC1-INH was administered following a single dose of icatibant. When data from the outliers were excluded, pdC1-INH use occurred in 8.7, 7.9, 10.9, and 12.0% of attacks for patients with underweight, normal, overweight, and obese BMI, respectively (P = 0.0232). Thus, there was a slight increase in the rate of pdC1-INH use with higher BMI.

**Table 3:** Treatment of attacks

	Underweight BMI	Normal BMI	Overweight BMI	Obese BMI
Type of administration, n (%)^a^				
n	103	1261	792	415
HCP	10 (9.7)	367 (29.1)	148 (18.7)	67 (16.1)
Self	93 (90.3)	894 (70.9)	644 (81.3)	348 (83.9)
No. of icatibant injections per attack^a^				
n	103	1301	826	434
Mean ± SD	1.0 ± 0.2	1.1 ± 0.3	1.1 ± 0.3	1.1 ± 0.4
Median (range)	1 (1–3)	1 (1–3)	1 (1–3)	1 (1–6)
No. of icatibant injections per attack, n (%)^a^				
n	103	1301	826	434
1	91 (88.3)	1090 (83.8)	687 (83.2)	312 (71.9)
1 + C1-INH rescue medication	9 (8.7)	83 (6.4)	85 (10.3)	73 (16.8)
2	2 (1.9)	112 (8.6)	48 (5.8)	24 (5.6)
2 + C1-INH rescue medication	0	8 (0.6)	5 (0.6)	22 (5.1)
3	1 (1.0)	6 (0.5)	1 (0.1)	2 (0.5)
3 + C1-INH rescue medication	0	2 (0.2)	0	0
6	0	0	0	1 (0.2)^b^
C1-INH rescue medication, n (%)				
n	104	1314	829	450
No. of attacks used C1-INH rescue	9 (8.7)	101 (7.7)^c^	90 (10.9)	96 (21.3)^c,d^
No. of patients used C1-INH rescue	2	29	21	16

*BMI* body mass index; *C1*-*INH* C1-inhibitor; *HCP* health care provider; *SD* standard deviation; n = number of attacks, excluding attacks with missing or unknown data

^a^Two patients (one normal BMI, one obese BMI) were found to be outliers because of an abnormally high rate of reinjections and rescue medication use. However, their data were included in this analysis

^b^One patient experienced an abdominal attack that lasted for 6 days; the patient was treated with one icatibant injection each day, for a total of six injections

^c^When data from the outlier patient were excluded, 47/393 (12.0%) of attacks were treated with C1-INH. The other outlier patient did not use any rescue medication (Additional file 1: Table S3)

^d^One attack was treated with C1-INH; however, the number of icatibant injections used was unknown

### Treatment outcomes

**Fig. 3:**
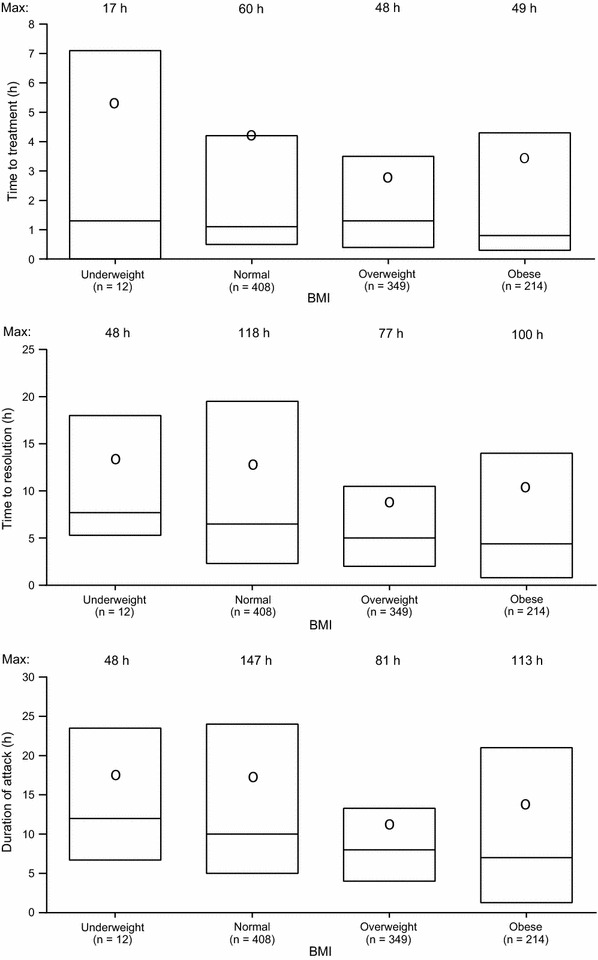
Outcomes of attacks treated with icatibant by body mass index (BMI). Analysis included attacks with data for all three outcomes. Boxes depict 25th percentile, median, and 75th percentile. Mean indicated with “○”. P values refer to comparisons versus normal BMI: time to treatment, P = 0.007 versus overweight and P = 0.385 versus obese; duration of attack, P < 0.001 versus overweight and P = 0.025 versus obese; time to resolution, P < 0.001 versus overweight and P = 0.021 versus obese. Results excluding data from the two reinjection outliers are presented in Additional file 1: Figure S3. Max = maximum value. n = number of attacks. Time (h) is base-10 log-transformed

Overall, there was no difference among the BMI groups in time to treatment (P = 0.468; Fig. 3). However, pair-wise comparisons showed that time to treatment was shorter for patients with overweight BMI compared with patients with normal BMI (P = 0.007). There were significant differences overall among the BMI groups with respect to duration of attack and time to resolution (P < 0.001 for both outcomes). Moreover, both outcomes were significantly shorter for patients with overweight and obese BMI compared with patients with normal BMI. Time to resolution was significantly extended in patients with overweight and obese BMI if they treated attacks ≥ 1 or ≥ 2 h after attack onset compared with earlier treatment (Table 4). However, this impact on time to resolution was not observed in patients with normal BMI.

**Table 4:** Impact of time to treatment on mean time to resolution and duration of attack

Time to treatment	n	Underweight BMI	n	Normal BMI	n	Overweight BMI	n	Obese BMI
Mean ± SD time to resolution								
0 to < 1 h	6	15.6 ± 16.7	155	10.4 ± 12.5	148	7.9 ± 11.0	108	5.4 ± 9.5
≥ 1 h	6	10.1 ± 8.4	253	15.0 ± 17.7	201	9.9 ± 12.4	106	15.0 ± 17.4
P value		n.a.		0.078		0.001		< 0.001
0 to < 2 h	6	15.6 ± 16.7	228	11.8 ± 13.5	193	8.4 ± 12.0	142	6.5 ± 10.1
≥ 2 h	6	10.1 ± 8.4	180	15.0 ± 18.6	156	9.8 ± 11.6	72	17.5 ± 19.2
P value		n.a.		0.734		0.011		< 0.001
Mean ± SD duration of attack								
0 to < 1 h	6	15.8 ± 16.7	155	10.7 ± 12.5	148	8.1 ± 11.0	108	5.6 ± 9.5
≥ 1 h	6	18.7 ± 12.5	253	21.8 ± 22.0	201	14.8 ± 14.2	106	22.4 ± 20.8
P value		n.a.		< 0.001		< 0.001		< 0.001
0 to < 2 h	6	15.8 ± 16.7	228	12.3 ± 13.7	193	8.9 ± 12.1	142	6.9 ± 10.2
≥ 2 h	6	18.7 ± 12.5	180	24.2 ± 23.8	156	15.8 ± 13.9	72	27.7 ± 22.1
P value		n.a.		< 0.001		< 0.001		< 0.001

Results excluding the two outlier patients are presented in Additional file 1: Table S4

*BMI* body mass index; *n.a.* not applicable; statistical comparison was not conducted due to small sample sizes; *SD* standard deviation; n = the number of attacks

Multivariate regression analyses showed that patients with BMI ≥ 25 kg/m^2^ were more likely to treat attacks within 1 h than patients with BMI < 25 kg/m^2^ (P < 0.0295; Table 5). Patients with a high frequency of attacks also were more likely to treat attacks early, and country also plays a role in time to treatment in the univariate analysis (P < 0.0001), which was not confirmed in the multivariate analysis (Table 5). Time to resolution was more likely to be shorter for patients with higher BMI, and for attacks that were treated with C1-INH rescue medication or that affected the skin (Table 6).

**Table 5:** Evaluation of factors affecting time to treatment^a^

Effect (numerator)	Odds ratio	95% CI	P value
Univariate analysis^b^			
Attack frequency (≥ 10 attacks/year)	2.48	–	< 0.001
BMI (≥ 25 kg/m^2^)	1.77	–	0.012
Type of administration (HCP)	0.55	–	0.067
Country			< 0.0001^c^
Multivariate analysis^d^			
Attack frequency (≥ 10 attacks/year)^e^	2.89	1.36–6.14	0.0056
BMI (≥ 25 kg/m^2^)^e^	1.71	1.06–2.79	0.0295

*BMI* body mass index; *CI* confidence interval; *HCP* health care provider

^a^Model of probability that time to treatment < 1 h

^b^Only effects with P < 0.2 are shown. Complete results are presented in Additional file 1: Table S5

^c^Overall effect of country on time to treatment

^d^Only significant effects are shown

^e^Results were similar when data from the two reinjection outliers were excluded (Additional file 1: Table S6)

**Table 6:** Evaluation of factors affecting time to resolution^a^

Effect (numerator)	Odds ratio	95% CI	P value
Univariate analysis^b^			
BMI (≥ 25 kg/m^2^)	1.52	–	0.072
C1-INH rescue medication (yes)	0.66	–	0.097
Affected site: skin (yes)	0.74	–	0.118
Type of administration (HCP)	1.49	–	0.133
Time to first injection (≥ 1 h)	0.78	–	0.142
Country			0.019^c^
Multivariate analysis^d^			
BMI (≥ 25 kg/m^2^)	4.46	2.24–8.89	< 0.0001
C1-INH rescue medication (yes)	0.31	0.19–0.50	< 0.0001
Affected site: skin (yes)	0.65	0.43–1.00	0.049

*BMI* body mass index; *C1*-*INH* C1-inhibitor; *CI* confidence interval; *HCP* health care provider

^a^Model of probability that time to resolution < 5 h

^b^Only effects with P < 0.2 are shown. Complete results are presented in Additional file 1: Table S7

^c^Overall effect of country on time to resolution

^d^Only significant effects are shown. Complete results excluding the two reinjection outliers are presented in Additional file 1: Table S8

### Adverse events (AEs)

There was no difference in the rate of AEs between patients with underweight, normal, overweight, and obese BMI (Table 7). The most common treatment-related AEs across all other BMI groups were injection site reactions such as injection site pain (one report in one patient with overweight BMI) and injection site erythema (six reports in one patient with overweight BMI and one report in one patient with normal BMI). There were no injection site reactions in patients with obese BMI. There were no differences between the BMI groups with respect to the rate of vascular AEs. Two patients in the overweight BMI group reported a total of three serious AEs related to icatibant (gastritis and reflux esophagitis in one patient and angioedema in another patient).

**Table 7:** AEs in all patients with C1-INH-HAE

	Underweight BMI (n = 18)	Normal BMI (n = 210)	Overweight BMI (n = 162)	Obese BMI (n = 73)
No. of patients, no. of events	3, 3	48, 101	33, 91	19, 53
AEs related to icatibant (no. of patients, no. of events)^a^	0, 0	7, 24	6, 29	2, 5
General disorders and administration site conditions	0, 0	5, 9	3, 11	1, 1
Vascular disorders	0, 0	3, 5	3, 4	1, 3
Skin and subcutaneous tissue disorders	0, 0	0, 0	2, 2	1, 1
Gastrointestinal disorders	0, 0	2, 2	1, 3	0, 0
Nervous system disorders	0, 0	1, 1	1, 2	0, 0
Investigations	0, 0	2, 5	0, 0	0, 0
Serious AEs (no. of patients, no. of events)	0, 0	20, 28	16, 32	11, 30
Serious AEs related to icatibant and (no. of patients, no. of events)^b^	0, 0	0, 0	2, 3	0, 0
Skin and subcutaneous tissue disorders				
Angioedema	0, 0	0, 0	1, 1	0, 0
Gastrointestinal disorders				
Gastritis	0, 0	0, 0	1, 1	0, 0
Reflux esophagitis	0, 0	0, 0	1, 1	0, 0

A missing relationship to icatibant was considered related to icatibant

*AE* adverse event; *BMI* body mass index; *C1*-*INH*-*HAE* hereditary angioedema due to C1-inhibitor deficiency; n = number of patients

^a^Listed by medical dictionary for regulatory activities system organ class. Only AEs that were reported in ≥ 2 patients are presented

^b^Listed by medical dictionary for regulatory activities system organ class and preferred term

## Discussion

The results of our analysis of real-world data showed that the frequency and characteristics of C1-INH-HAE attacks are generally similar across BMI groups. Interestingly, the rate of attacks according to site significantly differed by BMI group in that patients with high BMI reported fewer attacks on the abdomen and more attacks on the skin. The reason for the difference is unknown, but a relationship between BMI and mediators of angioedema or inflammation in the gut could be possible.

The results of our analysis of real-world observational data showed that treatment of C1-INH-HAE attacks with icatibant was successful regardless of BMI. The majority of attacks across BMI groups were treated with a single dose of icatibant and without the need for pdC1-INH rescue medication. Although there was a higher rate of pdC1-INH rescue medication use in patients with obese BMI (even when data from the two outlier patients were excluded), the difference between obese and other BMI groups was minimal. This suggests that overall, it is not necessary to adjust the administered dose of icatibant according to body weight.

Data from studies in healthy volunteers showed a significant correlation between body weight and the clearance and volume of distribution of icatibant, resulting in decreased systemic exposure for those with higher body weight [8]. This lower exposure could explain why there was an apparent increase in the rate of rescue medication use in patients with higher BMI. However, these studies were limited to volunteers with BMI < 30 kg/m^2^, thus effects in obese patients are unclear.

Patients with higher BMI were more likely to treat with icatibant within 1 h after the onset of an attack, and time to resolution and duration of attack were subsequently shorter in these patients. However, factors that impact BMI such as sex and age did not contribute to this outcome. The early treatment observed in overweight and obese BMI patients could be attributed to higher attack severity in these patients, or to the longer time required for attack resolution with delayed treatment in these groups. Earlier treatment and higher severity may suggest a more rapid onset of attacks in obese BMI patients, or conversely, a greater perception of symptoms leading to earlier treatment.

The results of the analysis presented here should be considered in the context that this was a retrospective analysis of real-world data rather than a randomized controlled clinical trial examining differences in the effectiveness of icatibant in patients with low or high BMI. Over half of the patients in this analysis had overweight or obese BMI, however this was similar to the overall distribution of BMI for adults in Europe [9]. Data collection was dependent on patient compliance with accurately documenting their attacks and treatments. In addition, the data collected on untreated attacks or attacks treated with other drugs were not as detailed as the data collected for icatibant-treated attacks. Thus, we were unable to fully evaluate the severity of disease in patients in the three BMI groups, as it is possible that not all attacks were accounted for.

## Conclusions

In conclusion, icatibant was well tolerated and used successfully to treat attacks in patients with overweight and obese BMI.

## Additional file

**Additional file 1.** Effectiveness of icatibant for treatment of hereditary angioedema attacks is not affected by body weight: findings from the Icatibant Outcome Survey, a cohort observational study.

## Abbreviations

AE: adverse event
BMI: body mass index
C1-INH: C1-inhibitor
C1-INH-HAE: HAE due to C1-inhibitor deficiency
CI: confidence interval
HCP: health care professional
IOS: Icatibant Outcome Survey
n.a.: not applicable
OR: odds ratio
SD: standard deviation
