# Supplementary material for: Effectiveness of icatibant for treatment of hereditary angioedema attacks is not affected by body weight: findings from the Icatibant Outcome Survey, a cohort observational study
Source: Clin Transl Allergy. 2018 Mar 23;8:11. doi: 10.1186/s13601-018-0195-x (PMC5870812; doi:10.1186/s13601-018-0195-x)
Supplement: Supplementary file 1 — Additional file 1. Effectiveness of icatibant for treatment of hereditary angioedema attacks is not affected by body weight: findings from the Icatibant Outcome Survey, a cohort observational study. [file 13601_2018_195_MOESM1_ESM.pdf]

Effectiveness of icatibant for treatment of hereditary angioedema attacks is not affected by body weight: findings from the Icatibant Outcome Survey, a cohort observational study

Supplemental Materials

Table S1. Patient BMI and number of icatibant-treated attacks (excluding 2 outlier patients)

Characteristic	Underweight BMI	Normal BMI	Overweight BMI	Obese BMI
Patients, n (%)	12	152	119	57
BMI, kg/m^{2}[a]				
Mean ± SD	18.0 ± 0.5	22.2 ± 1.8	26.9 ± 1.3	34.5 ± 4.1
Median (range)	18.1 (16.7–18.4)	22.5 (18.7–25.0)	26.6 (25.0–29.8)	33.3 (30.0–46.7)
No. of icatibant-treated attacks during enrollment	104	1271	829	393
No. of icatibant-treated attacks per patient[b]				
Mean ± SD	8.7 ± 13.5	8.4 ± 14.6	7.0 ± 11.3	6.9 ± 8.6
Median (range)	4.0 (1–47)	3.5 (1–101)	3.0 (1–83)	3.0 (1–33)

BMI body mass index; *SD* standard deviation

[a]At study entry

[b]Attack rate during enrollment. P = 0.510 comparing the normal, overweight, and obese categories. The underweight category was excluded from the comparison due to small sample size

Table S2. Treatment of attacks (excluding 2 outlier patients)

	Underweight BMI	Normal BMI	Overweight BMI	Obese BMI
Type of administration, n (%)				
n[a]	103	1218	792	358
HCP	10 (9.7)	367 (30.1)	148 (18.7)	67 (18.7)
Self	93 (90.3)	851 (69.9)	644 (81.3)	291 (81.3)
No. of icatibant injections per attack[a]				
n	103	1258	826	377
Mean ± SD	1.0 ± 0.2	1.1 ± 0.3	1.1 ± 0.3	1.1 ± 0.4
Median (range)	1 (1–3)	1 (1–3)	1 (1–3)	1 (1–6)
No. of icatibant injections per attack, n (%)[a]				
n	103	1258	826	377
1	91 (88.3)	1064 (84.6)	687 (83.2)	309 (82.0)
1 + C1-INH rescue medication	9 (8.7)	83 (6.6)	85 (10.3)	46 (12.2)
2	2 (1.9)	98 (7.8)	48 (5.8)	19 (5.0)
2 + C1-INH rescue medication	0	8 (0.6)	5 (0.6)	0
3	1 (1.0)	3 (0.2)	1 (0.1)	2 (0.5)
3 + C1-INH rescue medication	0	2 (0.2)	0	0
6	0	0	0	1 (0.3)[b]
C1-INH rescue medication, n (%)				
n	104	1271	829	393
No. of patients used C1-INH rescue	2	29	21	15
No. of attacks used C1-INH rescue	9 (8.7)	101 (7.9)	90 (10.9)	47 (12.0)[c]

BMI body mass index; *C1-INH* C1-inhibitor; *HCP* health care provider; *SD* standard deviation

n = number of attacks

[a]Excluding attacks with missing data

[b]One patient experienced an abdominal attack that lasted for 6 days; the patient was treated with one icatibant injection each day, for a total of six injections
[c]One attack was treated with C1-INH, however, the number of icatibant injections used was unknown

Table S3. Impact of time to treatment on mean time to resolution and duration of attack (excluding 2 outlier patients)

Time to treatment	n	Underweight BMI	n	Normal BMI	n	Overweight BMI	n	Obese BMI
Mean ± SD impact on time to resolution								
0 to < 1 h	6	15.6 ± 16.7	154	10.3 ± 12.5	148	7.9 ± 11.0	71	5.7 ± 10.9
≥ 1 h	6	10.1 ± 8.4	233	15.7 ±18.1	201	9.9 ± 12.4	88	16.2 ± 18.1
P value		n.a.		0.006		0.001		< 0.001
0 to < 2 h	6	15.6 ± 16.7	226	11.8 ± 13.6	193	8.4 ± 12.0	92	7.0 ± 11.7
≥ 2 h	6	10.1 ± 8.4	161	16.1 ± 19.2	156	9.8 ± 11.6	67	17.8 ± 19.2
P value		n.a.		0.174		0.011		< 0.001
Mean ± SD impact on duration of attack								
0 to < 1 h	6	15.8 ± 16.7	154	10.6 ± 12.5	148	8.1 ± 11.0	71	5.9 ± 10.9
≥ 1 h	6	18.7 ± 12.5	233	22.5 ± 22.5	201	14.8 ± 14.2	88	24.6 ± 21.4
P value		n.a.		< 0.001		< 0.001		< 0.001
0 to < 2 h	6	15.8 ± 16.7	226	12.4 ± 13.7	193	8.9 ± 12.1	92	7.4 ± 11.9
≥ 2 h	6	18.7 ± 12.5	161	25.4 ± 24.6	156	15.8 ± 13.9	67	28.4 ± 22.1
P value		n.a.		< 0.001		< 0.001		< 0.001

BMI body mass index; *n.a.* not applicable because statistical comparison was not conducted due to small sample sizes; *SD* standard deviation

Table S4. Evaluation of factors affecting time to treatment[a]

Effect (numerator)	Odds ratio	95% CI	P value
Univariate analysis			
Attack frequency (≥ 10 attacks/year)	2.48	–	< 0.001
BMI (≥ 25 kg/m^2)	1.77	–	0.012
Type of administration (HCP)	0.55	–	0.067
C1-INH long-term prophylaxis or rescue medication (yes)	1.28	–	0.289
Age at attack (≥ 40 years)	1.28	–	0.335
Severity (severe/very severe)	0.88	–	0.461
C1-INH rescue medication (yes)	1.19	–	0.466
Affected site: abdomen (yes)	0.93	–	0.582
Sex (female)	1.15	–	0.569
Affected site: skin (yes)	1.02	–	0.820
Affected site: larynx (yes)	1.05	–	0.821
Affected site: multiple sites (multiple)	1.03	–	0.902
C1-INH long-term prophylaxis (yes)	0.99	–	0.989
Country			< 0.0001[b]
Multivariate analysis			
BMI (≥ 25 kg/m^2)	1.71	1.06–2.79	0.0295
Attack frequency (≥ 10 attacks/year)	2.89	1.36–6.14	0.006
Attack frequency and type of administration[c]			
≥ 10 attacks/year self vs < 10 attacks/year HCP	5.81	1.08–31.28	0.035

BMI body mass index; *C1-INH* C1-inhibitor; *CI* confidence interval; *HCP* health care professional

[a]Model of probability that time to first injection < 1 hour

[b]Pr>ChiSq. Overall effect of country on time to treatment

[c]Only significant interactions are shown

Table S5. Evaluation of factors affecting time to treatment (excluding 2 outlier patients)[a]

Effect (numerator)	Odds ratio	95% CI	P value
Univariate analysis			
Attack frequency (≥ 10 attacks/year)	2.50	–	< 0.001
BMI (≥ 25 kg/m^2)	1.74	–	0.016
Type of administration (HCP)	0.56	–	0.077
C1-INH long-term prophylaxis (yes)	2.50	–	0.112
Age at attack (≥ 40 years)	1.26	–	0.370
Severity (severe/very severe)	0.87	–	0.449
C1-INH long-term prophylaxis or rescue medication (yes)	1.20	–	0.461
Sex (female)	1.20	–	0.460
C1-INH rescue medication (yes)	1.14	–	0.618
Affected site: abdomen (yes)	0.93	–	0.646
Affected site: larynx (yes)	1.06	–	0.804
Affected site: multiple sites (multiple)	1.05	–	0.829
Affected site: skin (yes)	1.00	–	0.981
Country			< 0.0001[b]
Multivariate analysis			
BMI (≥ 25 kg/m^2 vs < 25 kg/m^2)	1.69	1.03–2.76	0.037
Attack frequency (≥ 10 attacks/year vs < 10 attacks/year)	2.90	1.35–6.23	0.006
Attack frequency and type of administration[c]			
≥ 10 attacks/year self vs < 10 attacks/year HCP	6.03	1.09–33.31	0.033

BMI body mass index; *C1-INH* C1-inhibitor; *CI* confidence interval; *HCP* health care professional

[a]Model of probability that time to first injection < 1 hour

[b]Pr>ChiSq. Overall effect of country on time to treatment

[c]Only significant interactions are shown

Table S6. Evaluation of factors affecting time to resolution[a]

Effect (numerator)	Odds ratio	95% CI	P value
Univariate analysis			
BMI (≥ 25 kg/m^2)	1.52	–	0.072
C1-INH long-term prophylaxis or rescue medication (yes)	0.64	–	0.064
C1-INH rescue medication (yes)	0.66	–	0.097
Affected site: skin (yes)	0.74	–	0.118
Time to first injection (≥ 1 hour)	0.78	–	0.142
Type of administration (HCP)	1.49	–	0.133
Affected site: abdomen (yes)	1.27	–	0.257
Affected site: multiple sites (multiple)	0.81	–	0.264
Attack frequency (≥ 10 attacks/year)	1.26	–	0.322
Age at attack (≥ 40 years)	0.84	–	0.454
Severity (severe/very severe)	0.92	–	0.561
C1-INH long-term prophylaxis (yes)	0.73	–	0.550
Affected site: larynx (yes)	1.11	–	0.634
Sex (female)	1.08	–	0.738
Country			0.0191[b]
Multivariate analysis			
BMI (≥ 25 kg/m^2)	4.46	2.24–8.89	< 0.0001
C1-INH rescue medication (yes)	0.31	0.19–0.5	< 0.0001
Affected site: skin (yes)	0.65	0.43–1.00	0.0488
Type of administration (HCP)	1.54	0.92–2.59	0.1006
BMI and type of administration[c]			
≥ 25 kg/m^2 HCP vs < 25 kg/m^2 HCP	10.46	2.38–45.86	0.0002
≥ 25 kg/m^2 HCP vs ≥ 25 kg/m^2 self	3.62	1.36–9.64	0.0033
≥ 25 kg/m^2 HCP vs < 25 kg/m^2 self	6.88	2.12–22.39	0.0001
BMI and C1-INH rescue medication[c]			

≥ 25 kg/m^2 no vs < 25 kg/m^2 no	2.27	1.02–5.04	0.0398
≥ 25 kg/m^2 no vs < 25 kg/m^2 yes	14.38	4.83–42.83	< 0.0001
≥ 25 kg/m^2 yes vs < 25 kg/m^2 yes	8.77	2.22–34.67	0.0002
< 25 kg/m^2 no vs < 25 kg/m^2 yes	6.33	2.54–15.76	< 0.0001

BMI body mass index; *C1-INH* C1-inhibitor; *CI* confidence interval; *HCP* health care provider

[a]Model of probability that time to resolution < 5 hours

[b]Pr>ChiSq. Overall effect of country on time to resolution

[c]Only significant interactions are shown

Table S7. Evaluation of factors affecting time to resolution (excluding 2 outlier patients)[a]

Effect (numerator)	Odds ratio	95% CI	P value
Univariate analysis			
BMI (≥ 25 kg/m²)	1.57	–	0.053
Affected site: skin (yes)	0.69	–	0.060
C1-INH rescue medication (yes)	0.58	–	0.075
C1-INH long-term prophylaxis or rescue medication (yes)	0.60	–	0.078
Affected site: abdomen (yes)	1.39	–	0.114
Type of administration (HCP)	1.57	–	0.101
Time to first injection (≥ 1 hour)	0.87	–	0.379
Attack frequency (≥ 10 attacks/year)	1.23	–	0.373
Affected site: multiple sites (multiple)	0.87	–	0.446
Age at attack (≥ 40 years)	0.85	–	0.492
Affected site: larynx (yes)	1.12	–	0.561
Sex (female)	1.06	–	0.802
Severity (severe/very severe)	0.97	–	0.836
C1-INH long-term prophylaxis (yes)	0.97	–	0.973
Country			0.0174[b]
Multivariate analysis			
BMI (≥ 25 kg/m²)	2.50	1.38–4.53	0.0024
Type of administration (HCP)	1.53	0.92–2.54	0.1044
BMI and type of administration[c]			
≥ 25 kg/m² HCP vs < 25 kg/m² HCP	5.90	1.56–22.40	0.0027
≥ 25 kg/m² HCP vs ≥ 25 kg/m² self	3.60	1.31–9.89	0.0050
≥ 25 kg/m² HCP vs < 25 kg/m² self	3.82	1.32–11.05	0.0053

BMI body mass index; *C1-INH* C1-inhibitor; *CI* confidence interval; *HCP* health care provider

[a]Model of probability that time to resolution < 5 hours

[b]Pr>ChiSq. Overall effect of country on time to resolution

[c]Only significant interactions are shown

Table S8. Average duration of untreated attacks (excluding 2 outlier patients)

	Underweight BMI	Normal BMI	Overweight BMI	Obese BMI
Baseline				
Average duration of attack (hours)				
n	12	149	106	40
Mean ± SD[a]	50.7 ± 22.9	40.4 ± 32.5	45.9 ± 31.5	43.9 ± 34.9
Median (range)	48.0 (6–92)	40.0 (0–140)	48.0 (0–156)	44.1 (0–120)
Follow-up				
Average duration of attack (hours)				
n	11	76	70	25
Mean ± SD[a]	34.6 ± 21.6	39.7 ± 31.1	37.6 ± 30.6	44.2 ± 29.9
Median (range)	36 (0–72)	37.5 (0–144)	28.7 (0–120)	48.0 (0.3–120)

BMI body mass index; *SD* standard deviation

n = the number of patients

[a]P values comparing average duration of attack at baseline between patients with normal/overweight/obese BMI: P = 0.429 at baseline, P = 0.530 at follow-up

Note: average duration of untreated attacks corresponds to mean of average durations of untreated attacks at the skin, abdomen, larynx, and other sites.

Figure legends

Figure S1. Severity of icatibant-treated attacks by body mass index (BMI). P values comparing severity of attacks (very mild/mild/moderate vs severe/very severe): P = 0.146 for normal vs overweight; P = 0.972 for normal vs obese; P = 0.249 for overweight vs obese. n = number of attacks

Figure S2. Site of icatibant-treated attacks by body mass index (BMI). P values comparing frequency of attacks between patients with normal/overweight/obese BMI: P = 0.026 for skin attacks; P = 0.004 for abdominal attacks; P = 0.215 for laryngeal attacks; P = 0.142 for attacks affecting other organs. n = number of attacks

Figure S3. Outcomes of attacks treated with icatibant by body mass index (BMI). Analysis included attacks with data for all three outcomes. Boxes depict 25th percentile, median, and 75th percentile. Mean indicated with "○". P values refer to comparisons *versus* normal BMI: time to treatment, P = 0.016 *versus* overweight and P = 0.496 *versus* obese; duration of attack, P < 0.001 *versus* overweight and P = 0.417 *versus* obese; time to resolution, P < 0.001 *versus* overweight and P = 0.178 *versus* obese. Max = maximum value. n = number of attacks

Figure S1.

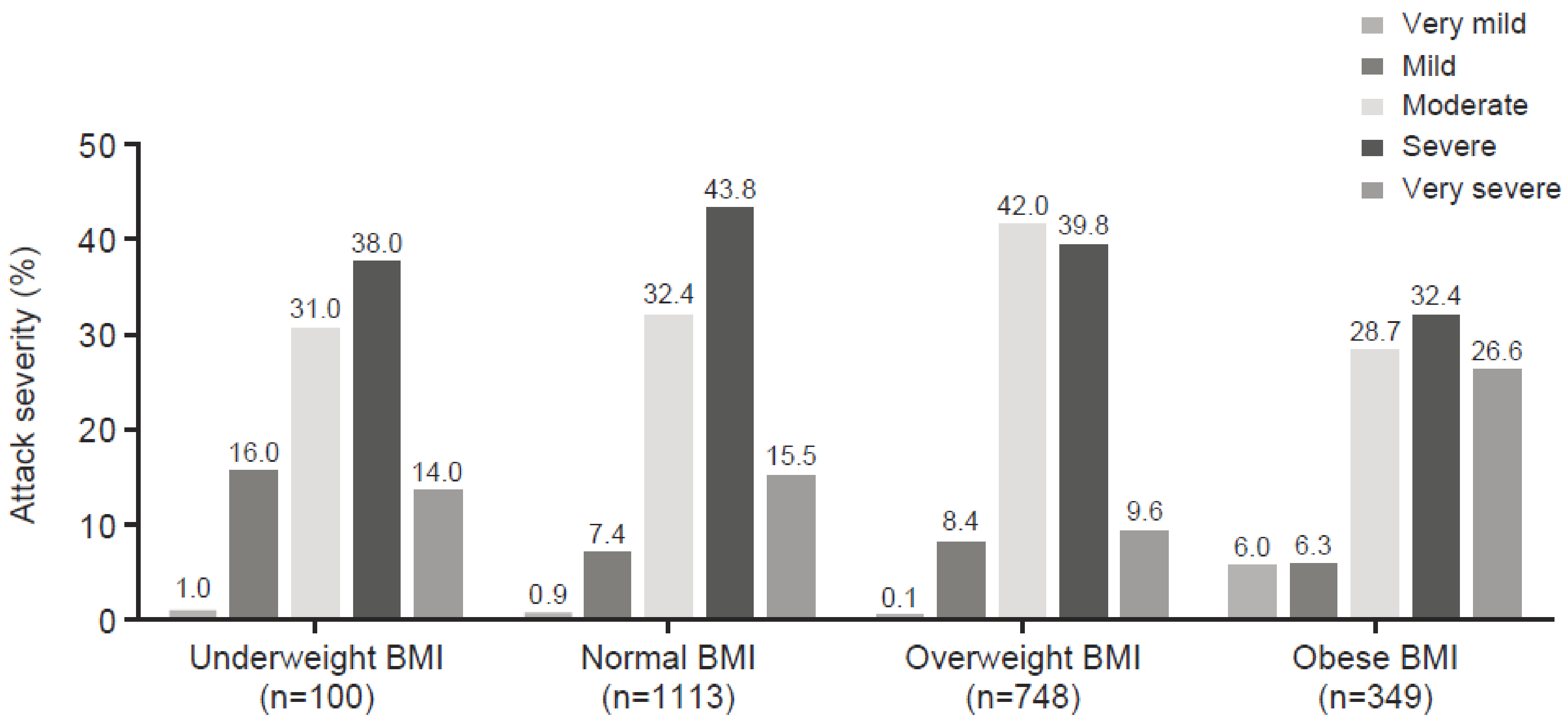
Very mild
Mild
Moderate
Severe
Very severe
Attack severity (%)
50
40
30
20
10
0
1.0
16.0
31.0
38.0
14.0
0.9
7.4
32.4
43.8
15.5
0.1
8.4
42.0
39.8
9.6
6.0
6.3
28.7
32.4
26.6
Underweight BMI
(n=100)
Normal BMI
(n=1113)
Overweight BMI
(n=748)
Obese BMI
(n=349)

Figure S2.

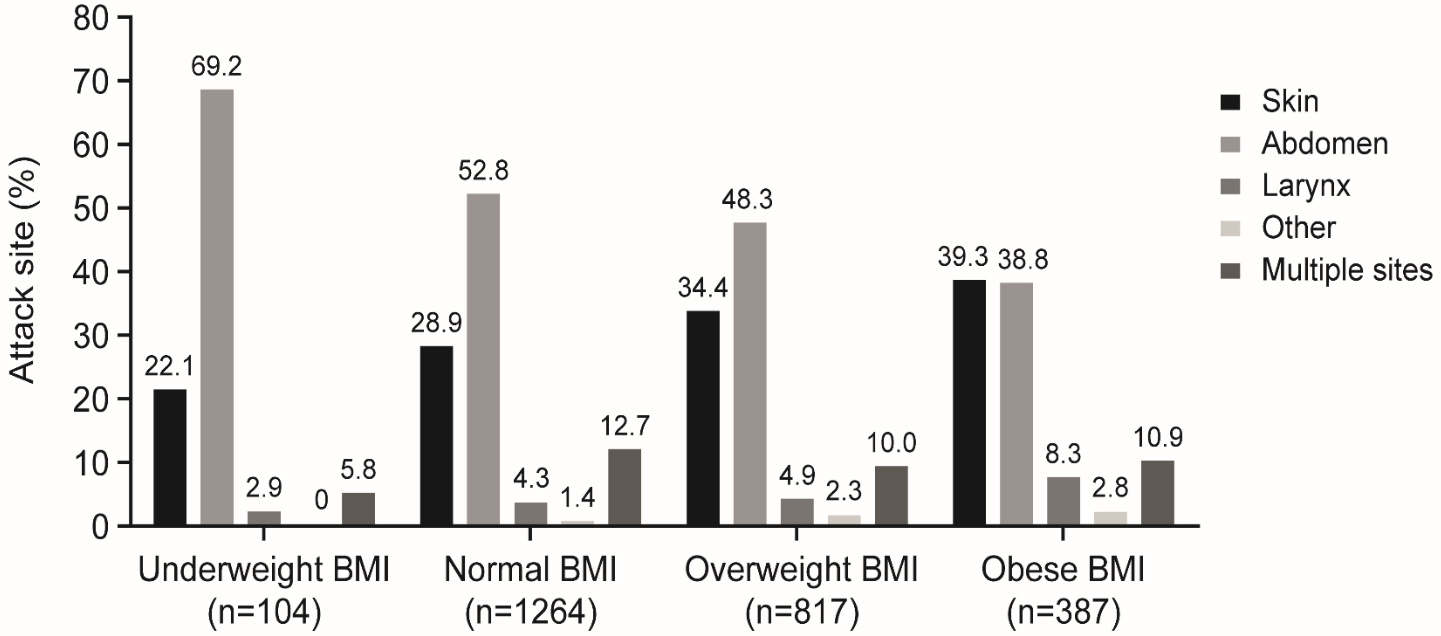
Attack site (%)
80
70
60
50
40
30
20
10
0
69.2
22.1
2.9
0
5.8
52.8
28.9
4.3
1.4
12.7
48.3
34.4
4.9
2.3
10.0
39.3
38.8
8.3
2.8
10.9
Underweight BMI (n=104)
Normal BMI (n=1264)
Overweight BMI (n=817)
Obese BMI (n=387)
Skin
Abdomen
Larynx
Other
Multiple sites

Figure S3.

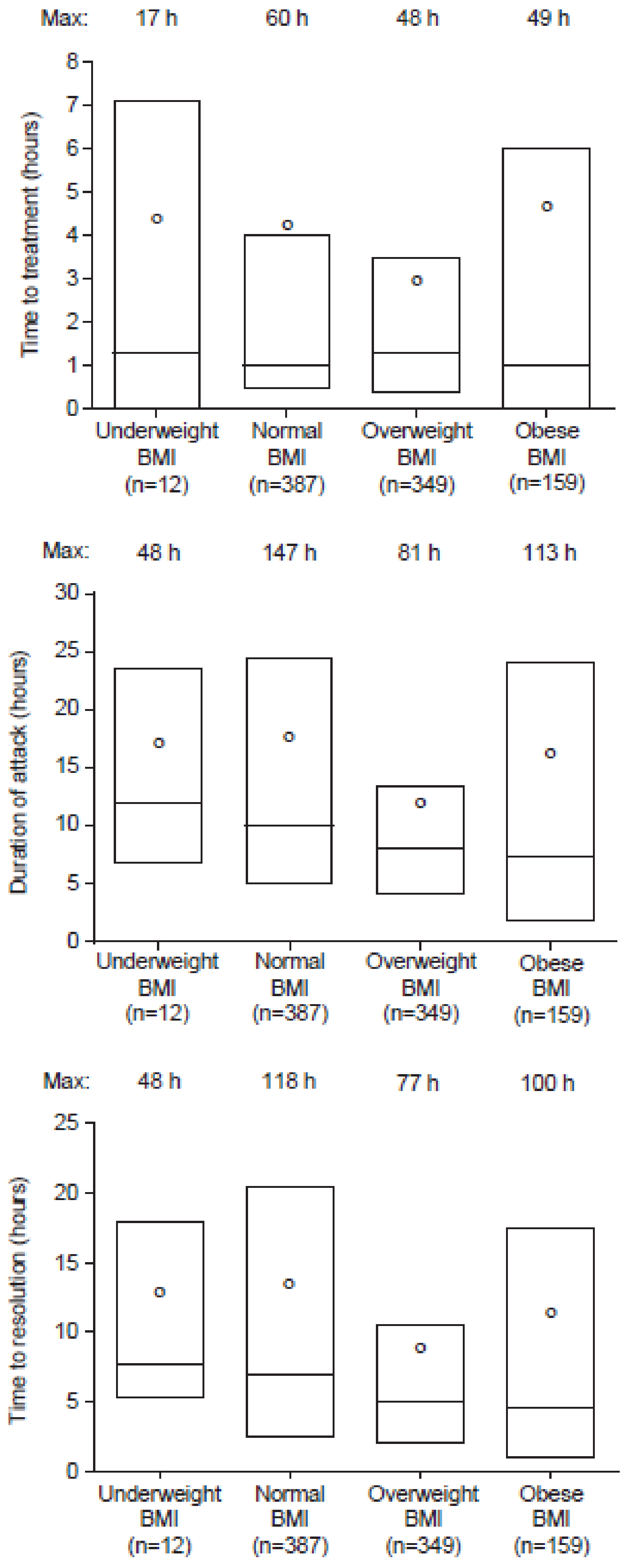
Max: 17 h 60 h 48 h 49 h
Time to treatment (hours)
0 1 2 3 4 5 6 7 8
Underweight BMI (n=12)
Normal BMI (n=387)
Overweight BMI (n=349)
Obese BMI (n=159)
Max: 48 h 147 h 81 h 113 h
Duration of attack (hours)
0 5 10 15 20 25 30
Underweight BMI (n=12)
Normal BMI (n=387)
Overweight BMI (n=349)
Obese BMI (n=159)
Max: 48 h 118 h 77 h 100 h
Time to resolution (hours)
0 5 10 15 20 25
Underweight BMI (n=12)
Normal BMI (n=387)
Overweight BMI (n=349)
Obese BMI (n=159)
